# Antioxidant Properties of a Traditional Vine Tea, *Ampelopsis grossedentata*

**DOI:** 10.3390/antiox8080295

**Published:** 2019-08-09

**Authors:** Kun Xie, Xi He, Keyu Chen, Jihua Chen, Kozue Sakao, De-Xing Hou

**Affiliations:** 1 Biological Science and Technology, United Graduate School of Agricultural Sciences, Kagoshima University, Kagoshima 890-0065, Japan; 2 College of Animal Science and Technology, Hunan Agricultural University, Changsha 410128, China; 3 Xiangya School of Public Health, Central South University, Changsha 410128, China; 4 Department of Food Science and Biotechnology, Faculty of Agriculture, Kagoshima University, Kagoshima 890-0065, Japan

**Keywords:** *Ampelopsis grossedentata*, dihydromyricetin, antioxidant ability, Nrf2/Keap1

## Abstract

*Ampelopsis grossedentata*, also called vine tea, has been used as a traditional beverage in China for centuries. Vine tea contains rich polyphenols and shows benefit to human health, but the chemical and antioxidant properties of vine tea polyphenols from different locations remain unclear. This study aims to investigate the chemical and antioxidant properties of vine tea from three major production areas in China including Guizhou, Hunan, and Guangxi Provinces. The highest amount of polyphenol from vine tea was extracted by 70% ethanol at 70 °C for 40 min with ultrasonic treatment. The major compound in vine tea polyphenols (VTP) was determined as dihydromyricetin (DMY) by high-performance liquid chromatography (HPLC) and the content was estimated as 21.42%, 20.17%, and 16.47% of dry weight basis from Hunan, Guizhou, and Guangxi products, respectively. The antioxidant activities were investigated *in vitro* and in culture hepatic cells. VTP and DMY showed strong 1,1-Diphenyl-2-picrylhydrazyl free radical (DPPH) scavenging ability and high oxygen radical absorption capacity (ORAC) value *in vitro*. VTP and DMY also increased the level of nicotinamide adenine dinucleotide phosphate (NADPH):quinone oxidoreductase (NQO1) in HepG2 cells. Moreover, VTP and DMY enhanced the level of nuclear factor erythroid 2-related factor 2 (Nrf2) and reduced the level of Kelch-like ECH-associated protein 1 (Keap1). Taken together, our data demonstrated that the extraction of vine tea by 70% ethanol with ultrasonic treatment is a novel method to efficiently obtain components possessing stronger antioxidant activity. Furthermore, the results from the culture cells suggest that the bioactive component of vine tea might exert the antioxidant activity by activating the cellular Nrf2/Keap1 pathway.

## 1. Introduction

Polyphenols are natural substances occurring in fruits, vegetables, beverages, and essential oils. These compounds can protect plants from oxidative stress and insects, and maintain the bioactivity in plant-derived food for humans. Plant polyphenols as antioxidant agents are now used to keep the properties of food in the aspects of both preservation and nutrition. Furthermore, dietary polyphenols intake has been linked to a lowered risk of the most common chronic diseases that are known to be caused by oxidative stress [1,2].

Oxidative stress is an imbalance status in oxidants and antioxidants, and is considered as a major factor in the pathogenesis of chronic disease [3,4]. The proper level of reactive oxygen species (ROS) in our body in a low-moderate concentration has positive effects such as involvement in energy production, regulation of cell growth, and intercellular signaling [5]. On the other hand, excess ROS can attack lipids in cell membranes, proteins in tissues or enzymes, and DNA to cause oxidation, which leads to lipid peroxidation and DNA damage [6]. This oxidation damage is considered to be an important factor of aging and aging-associated disease such as heart disease, cognitive dysfunction, and cancer [7]. A number of polyphenolic compounds have been reported to possess antioxidant properties in human study, and enhance the expression of cellular antioxidant enzymes through the nuclear factor erythroid 2-related factor 2 (Nrf2)-mediated pathway [8,9]. These activities account for the disease-preventing effects of polyphenol diets. Epidemiological studies have revealed an inverse correlation between the intake of fruits, vegetables, wine, tea, and the incidence of certain cancers and cardiovascular disease [10]. It has been reported that dietary polyphenols enhanced the function of antioxidant vitamins and enzymes to defend against the oxidative stress caused by excess ROS [11]. Thus, it is now well recognized that a daily intake of polyphenols in the diet is important for preventing some chronic diseases.

Green tea and black tea are the most consumed beverages worldwide, and their antioxidant properties are well investigated [12,13,14]. On the other hand, some traditional or folk teas from various edible plant leaves are also popular in Asia [15,16,17]. *Ampelopsis grossedentata*, also called vine tea, is a traditional herb widely used in medicine and health supplements in the southwest of China. The traditional manufacturing process of vine tea is similar to green tea, and people also usually drink vine tea by soaking it in boiling water as a health beverage. However, the growing environment of vine tea is different, and there is no standard for manufacturing process. Recently, it has been reported that the antioxidant capacity and major polyphenol composition of teas are affected by geographical location, plantation elevation, and leaf grades [18]. To clarify whether the antioxidant capacity and major polyphenol composition of vine tea are affected by the geographical locations and plantation elevation, we chose vine tea samples from three principle producing areas in China including Guizhou, Hunan, and Guangxi Provinces in this study. First, we used different solvents to optimize an efficient extraction method to obtain the highest content of polyphenol, and the major compounds in vine tea were then determined by high-performance liquid chromatography (HPLC). Second, we estimated the antioxidant capacity of the vine tea polyphenol (VTP) extract and its major compound, dihydromyricetin (DMY), from the above three locations. Finally, we used a culture cell line, HepG2, to investigate their antioxidant mechanisms in culture cells, focusing on their effect on the expression of Nrf2/Kelch-like ECH-associated protein 1 (Keap1)-mediated antioxidant enzymes.

## 2. Material and Methods

### 2.1. Samples and Chemical Reagents Preparation

The dried leaves and stems of vine tea were purchased from Hunan, Guizhou, and Guangxi Provinces, P.R. China. The gallic acid standard (3,4,5-trihydroxybenzoic acid, CAS Number 5995-86-8, purity ≥ 98%) and 6-hydroxy-2,5,7,8 tetramethyl chroman-2-carboxylic acid (Trolox) were purchased from Sigma-Aldrich (St. Louis, MO, USA). Dihydromyricetin (DMY) (CAS Number 27200-12-0, purity ≥ 98%) standard was purchased from Yuanye Bio-Technology Co. Ltd (Shanghai, China). Ethanol, methanol, acetone, and ethyl acetate were purchased from Sinopharm Chemical Reagent Co. Ltd (Shanghai, China). Phosphoric acid (HPLC purity) and methanol (HPLC purity) were obtained from Sigma (St. Louis, MO, USA). Fetal bovine serum (FBS) was obtained from Equitech-Bio (Kerrville, TX, USA). The antibodies against Nrf2, Keap1, Heme oxygenase 1 (HO-1), nicotinamide adenine dinucleotide phosphate (NADPH): quinone oxidoreductase (NQO1), and β-actin were from Santa Cruz Biotechnology (Santa Cruz, CA, USA).

### 2.2. Extraction and Total Polyphenol Content Analysis 

**Figure 1:**
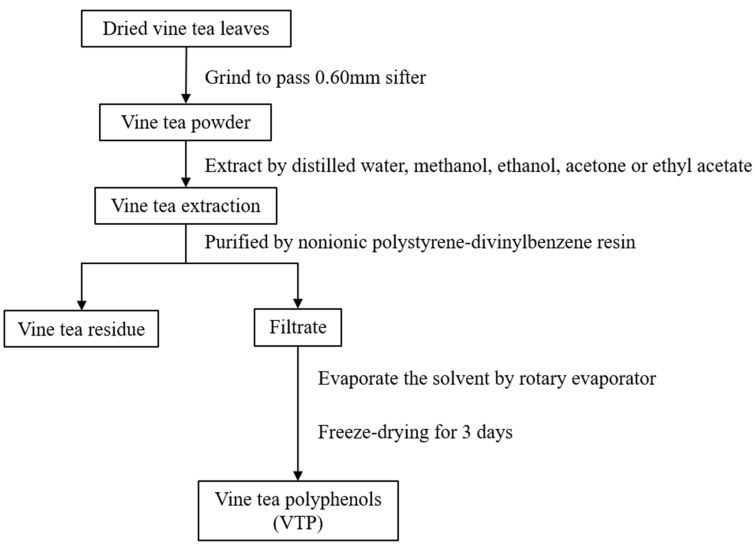
Diagram of vine tea polyphenol (VTP) extraction. All vine tea leaves from different locations were freeze-dried to ensure the same moisture content.

Vine tea was comminuted by a grinder to pass a 0.60 mm sifter and stored in a −20 °C freezer for further analysis. One gram of vine tea dry powder extracted with different solvents was placed in glass tubes and a condenser pipe was connected to the glass tubes to prevent solvent evaporation. The tubes were in a thermostatic water bath set at 15 °C in a fume hood, and the ratio of vine tea to solvent was 1:5. It has been reported that the extracting solvents significantly affected the total polyphenol content and antioxidant activity of the green tea extracts [19]. Thus, we used distilled water, ethanol, methanol, acetone, and ethyl acetate as the extracting solvents, and further investigated the effect of solvent concentration, extraction time, and temperature on the extraction efficiency, respectively. The extracts were filtrated with a 0.45 μm organic filter and concentrated by a rotary evaporator. The concentrated extracts were further purified by nonionic polystyrene-divinylbenzene resin, and freeze-dried for three days. The powder obtained was used as VTP (Figure 1).

The total polyphenol content was determined by the Folin–Ciocalteu method [20]. In brief, gallic acid (3,4,5-trihydroxybenzoic acid) standards were set at 1, 0.5, 0.25, 0.125, and 0.06125 mg/mL. Vine tea extracts were prepared at 0.5 mg/mL. One hundred microliters of vine tea extracts and standards were diluted with 900 μL distilled water in a 10 mL tube, 4 mL 0.2 N Folin and Ciocalteu’s phenol reagent (Sigma-Aldrich, Shanghai, China.), and 4 mL of 10% sodium carbonate aqueous solution were added into the tube. The tube was placed in a thermostat water bath set at 25 °C, the absorbance of each reaction was measured at 760 nm by a spectrophotometer (Thermo Fisher scientific, Oulu, Finland) after 2 h. Results are expressed as mg gallic acid equivalents (GAE) per g dry-matter of vine tea.

### 2.3. HPLC Analysis

HPLC analysis of VTP was performed in a Jasco MD-2015Plus HPLC (JASCO International Co. Ltd., Tokyo, Japan) equipped with a Cosmocore 2.6 C18 Packed Column (Nacalai Tesque Inc., Kyoto. Japan). Mobile phase A was 0.1% (*v/v*) phosphoric acid (aqueous), and mobile phase B was acetonitrile. A mobile phase consisting of 85% A and 15% B was delivered to the column at a flow rate of 1.00 mL/min at 27 °C. DMY was prepared at 2, 1, 0.5, 0.25, 0.125, and 0.06125 mg/mL in methanol. VTP extracted from different locations were prepared at 1 mg/mL in methanol. The injection volume was set at 10 μL. UV absorption spectra were obtained from 200 nm to 400 nm, and in particular, the absorbance at 290 nm was recorded.

### 2.4. Assay of 2,2-diphenyl-1-picrylhydrazy (DPPH) Free Radical Scavenging Activity

The radical scavenging activity of different locations of VTP and dihydromyricetin were measured by the DPPH method [21]. All of the VTP and DMY samples were prepared at 12.5, 25, 50, 100, 200, and 400 μg/mL in 70% ethanol. Trolox standards were prepared at 12.5, 25, 50, 100, 200, and 400 μM in 70% ethanol. Briefly, ten microliters of each sample were mixed with 190 μL of 0.2 mM DPPH in 96-well plates, and the final concentrations of VTP samples and DMY were 0.625, 1.25, 2.5, 5, 10, and 20 μg/mL. The absorbance was then measured at 492 nm with a microplate reader (Thermo scientific Multiscan FC, Tokyo, Japan) after the plate covered with aluminum foil was left for 30 min at 25 °C. The percentage scavenging rate of DPPH was calculated according to the formula:

DPPH Scavenging rate = (A0 − As)/A0 × 100%

where A0 represents the absorption of the blank sample, and as represents the absorption of VTP or other standards.

### 2.5. Assay of Oxygen Radical Absorbance Capacity (ORAC)

ORAC was measured according to the method as described previously [22]. In brief, one hundred microliters of fluorescein (7.5 nM), 10 μL of Trolox or VTP or DMY were added in the 96-well plate and incubated at 37 °C for 15 min. After the incubation, 40 μL of 2,2'-Azobis(2-amidinopropane) dihydrochloride (AAPH) (100 mM) was added rapidly to start the reaction, and the microplate was automatically shaken prior to each reading. The fluorescence was recorded every 2 min by a multilabel counter (PerkinElmer Co. Ltd., Tokyo, Japan). The ORAC values were calculated based on the area under curve (AUC) of the sample standardized by blank and Trolox standards. The data were expressed as Trolox equivalents (μmol TE/g).

### 2.6. Cell Culture and Western Blot Analysis

Human hepatoblastoma HepG2 cells obtained from the Cancer Cell Repository (Tohoku University, Sendai, Japan) were cultured in Dulbecco’s modified Eagle’s medium (DMEM) containing 10% FBS at 37 °C in a 5% CO_2_ atmosphere. HepG2 cells (5 × 10^5^ cells/dish) were precultured in 10 cm culture dishes for 24 h and then treated with various concentrations of VTP and DMY in 0.1% dimethyl sulfoxide (DMSO), and the control group was 0.1% DMSO only. The cells were harvested with modified RIPA buffer (50 mM Tris-HCl (pH 8.0), 150 mM NaCl, 1 mM EDTA, 1% Nonidet P-40, 0.25% Na-deoxycholate, 1 mM sodium fluoride, 1 mM sodium orthovanadate, 1 mM phenylmethylsulfonyl fluoride) plus proteinase inhibitor cocktail (Nacalai Tesque, Inc., Kyoto, Japan). Equal amounts of lysate protein were separated on sodium dodecyl sulfate-polyacrylamide gel electrophoresis (SDS-PAGE) and transferred to a polyvinylidene difluoride (PVDF) membrane electrophoretically (GE Healthcare UK Ltd., Amersham, England). After the membrane was blocked with TBST buffer (500 mM NaCl, 20 mM Tris-HCl (pH 7.4), and 0.1% Tween 20) containing 5% non-fat dry milk, the membrane was incubated overnight with the primary antibodies (β-actin, Nrf2, HO-1, and NQO1) at 4 °C and further incubated with HRP-conjugated secondary antibodies for another 1 h. The target proteins were detected using the enhanced chemiluminescence (ECL) system. The relative amounts of proteins bound with a specific antibody were quantified with Lumi Vision Imager software (TAITEC Co. Ltd., Saitama, Japan).

### 2.7. Statistical Analysis

The experiment results were presented as mean ± SD. The statistical differences between groups were performed by one-way analysis of variance tests, followed by Fisher’s least significant difference (LSD) and Duncan’s multiple range tests with the SPSS statistical program (version 19.0, IBM Corp., NY, USA.). A probability of *p* < 0.05 was considered as significant.

## 3. Results

### 3.1. Extraction Conditions for Vine Tea Polyphenol 

**Figure 2:**
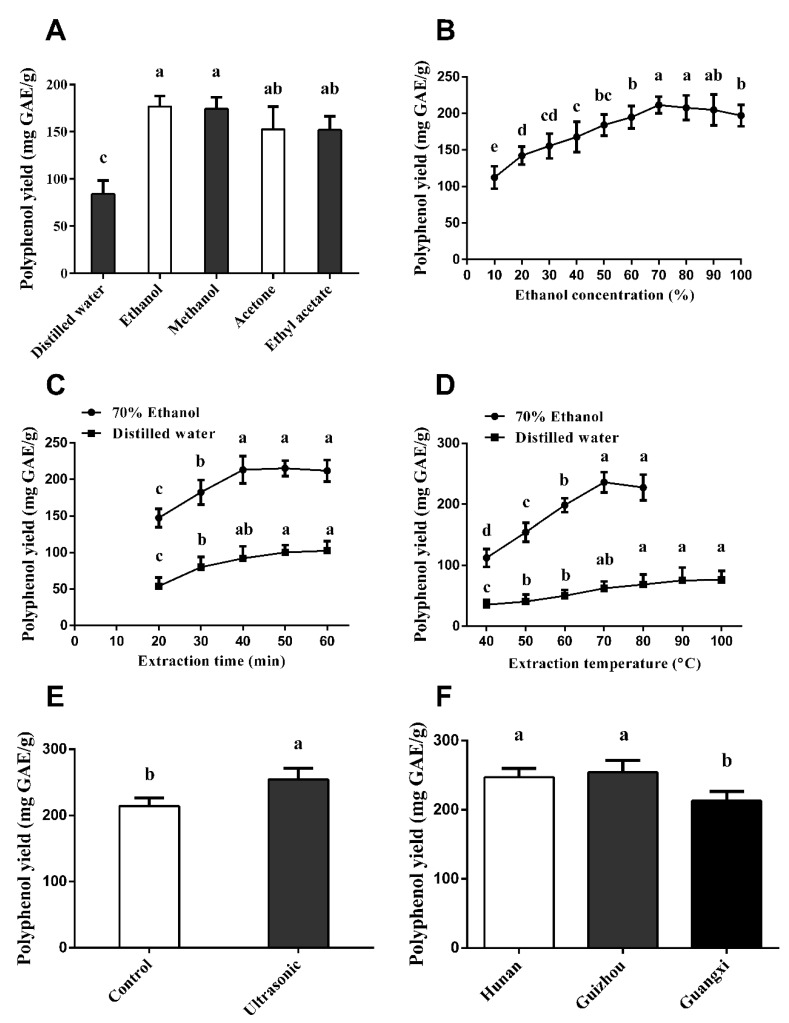
Conditions for extracting the vine tea polyphenols. (**A**) Polyphenol yield extracted by different solvents. (**B**) Polyphenol yield extracted by different concentrations of ethanol. (**C**) Polyphenol yield extracted by 70% ethanol with different times. (**D**) Polyphenol yield extracted by 70% ethanol at different temperatures. (**E**) Polyphenol yield extracted by 70% ethanol alone (control) and 70% ethanol plus ultrasonic treatment (ultrasonic). (**F**) Polyphenol yield of vine tea from different locations. The data represent mean ± SD with three repeats, and different letters in the same column indicate significant differences (*p* < 0.05).

As shown in Figure 2A, the extracts obtained by organic solvents including ethanol, methanol, acetone, and ethyl acetate showed significant higher polyphenol content than that by water (*p* < 0.05). Due to the use of ethanol being recognized in the food industry, we chose ethanol as the solvent to extract polyphenol from vine tea in this study. To optimize the efficiency conditions, the ethanol concentration, extraction time, and temperature were further investigated. The polyphenol yield was increased in a concentration-dependent manner from 10–70% ethanol, and the highest yield of polyphenol was obtained by 70% ethanol extraction (Figure 2B). The polyphenol yield was also observed in the extraction time and temperature-dependent manner from 20–60 min at 40–100 °C, respectively. Extraction with 70% ethanol at 70 °C for 40 min yielded the highest VTP (Figure 2C,D).

An ultrasonic extraction technique was reported to increase the polyphenol contents extracted from tea [23]. Our data also revealed that extraction plus ultrasonic treatment in the above conditions could significantly increase the yield of VTP than that by ethanol alone (*p* < 0.05, Figure 2E). Finally, we used these optimized extraction conditions to extract the polyphenols from three locations. As shown in Figure 2F, the total polyphenol contents of vine tea from Hunan and Guizhou Provinces were significantly higher than that from Guangxi Province (*p* < 0.05).

### 3.2. DMY Determination in Vine Tea Polyphenol by HPLC

**Figure 3:**
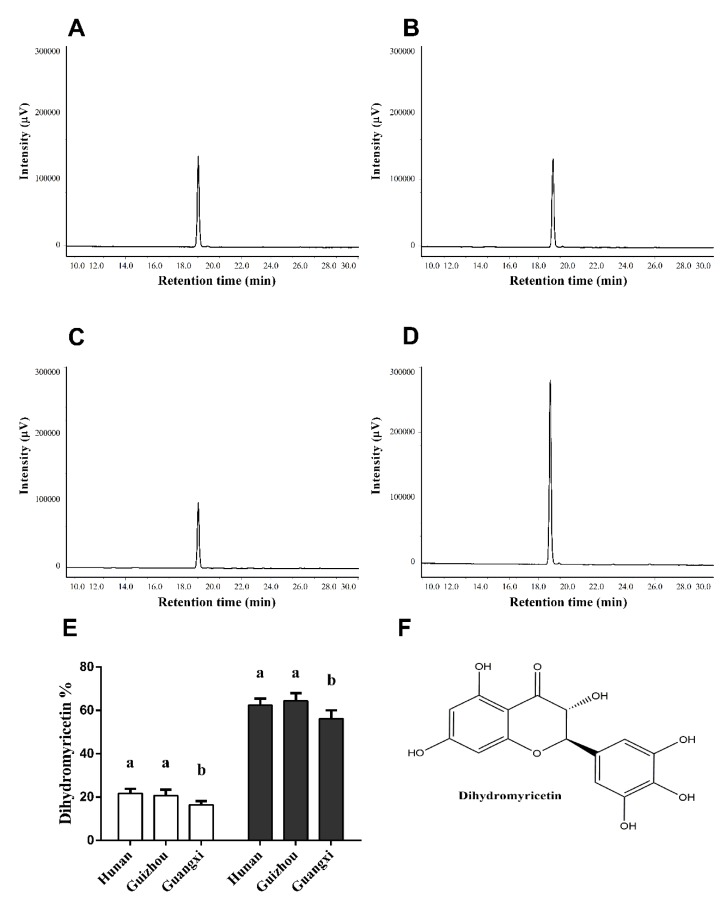
Dihydromyricetin determination in vine tea polyphenols by high-performance liquid chromatography (HPLC). The HPLC profiles of vine tea polyphenols (VTP) from Hunan Province (**A**), Guizhou Province (**B**), Guangxi Province (**C**) as well as the VTP plus standard dihydromyricetin (DMY) (**D**). (**E**) DMY content in the vine tea dry powder (white bar) and in VTP (black bar) from the above three locations. (**F**) Chemical structure of DMY. The data represent mean ± SD from three repeats, different letters in the same column indicate significant differences (*p* < 0.05).

It has been reported that the main component of vine tea is DMY [24], therefore, we determined DMY in VTP by HPLC with a standard DMY. Figure 3A–C show the HPLC profile with a main peak from Hunan, Guizhou, and Guangxi Provinces, respectively. Figure 3D shows the HPLC profile of a mixture containing 1 mg VTP and 1 mg standard DMY, where the main peak increased to about twice as high than that in VTP alone. These data indicate that the main peak in VTP is DMY. Furthermore, we estimated that the DMY content in vine tea came from three different locations, according to the dihydromyricetin standard curve. The DMY content was estimated as 21.67%, 20.79%, and 16.42% in the dry powder of vine tea (white bar, Figure 3E), and as 64.44%, 62.36%, and 56.22% in the vine tea polyphenol (VTP) extract (black bar, Figure 3E) from Hunan, Guizhou, and Guangxi Provinces respectively (Figure 3E). The DMY content from Guizhou and Hunan Provinces was significantly higher than that from Guangxi Province (*p* < 0.05).

### 3.3. DPPH Radical Scavenging Activity and ORAC Values

**Figure 4:**
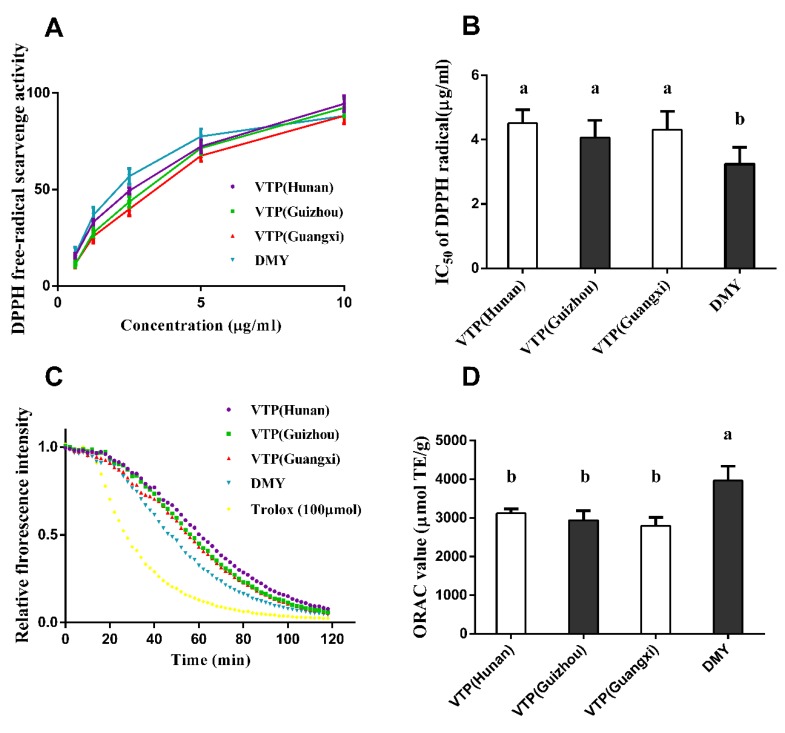
Antioxidant ability of VTP. (**A**) 2,2-diphenyl-1-picrylhydrazy (DPPH) free-radical scavenging rate of VTP from three locations and DMY. (**B**) IC_50_ value of the DPPH free-radical scavenging of VTP from three locations and DMY. (**C**) Relative florescence intensity of oxygen radical absorbance capacity (ORAC) of VTP from three locations and DMY in 2 h. (**D**) ORAC value (Trolox equivalent/g sample) of VTP from three locations and DMY. The data represent mean ± SD with three repeats, different letters in the same bar indicate significant differences (*p* < 0.05).

The DPPH radical is one of the few stable organic nitrogen radicals and can be simple and accurately measured [25]. Thus, we first used the DPPH assay to screen the radical scavenging activity of vine tea extract from three different locations. As shown in Figure 4A, a concentration-dependent manner was observed in the range of 0.6125 to 10 μg/mL. The concentration for scavenging 50% DPPH radicals (IC_50_) by VTP from Hunan, Guizhou, and Guangxi products were estimated as 4.51 μg/mL, 4.06 μg/mL, and 4.31 μg/mL, respectively. Moreover, the IC_50_ of pure DMY was estimated as 3.24 μg/mL, which was 0.7-fold of the IC_50_ than that of the VTP and significantly lower (Figure 4B). As we measured above, the DMY content was as high as 64.44%, 62.36%, and 56.22% in VTP from three locations. These data indicated that DMY is a major DPPH radical scavenger in VTP. The ORAC assay utilizes a controllable source of peroxyl radicals that can stimulate the antioxidant reactions with lipids in both food and physiological systems, which cannot be assayed by the DPPH assay [25]. Therefore, we further used the ORAC assay to evaluate the oxygen radical absorbance capacity of vine tea from three different locations. As shown in Figure 4C,D, the ORAC value of vine tea from Hunan, Guizhou, and Guangxi Provinces were estimated as 3116.97, 2941.61, and 2791.32 μmol of Trolox equivalent (TE) /g, respectively. No significant difference in ORAC value was observed between three products VTP (*p* > 0.05). The ORAC value of DMY was also significantly higher than that of VTP from three locations (*p* < 0.05), indicating DMY is also a major compound for ORAC in VTP.

### 3.4. Effect of VTP and DMY on Expression of Antioxidant Enzymes in HepG2 Cells

**Figure 5:**
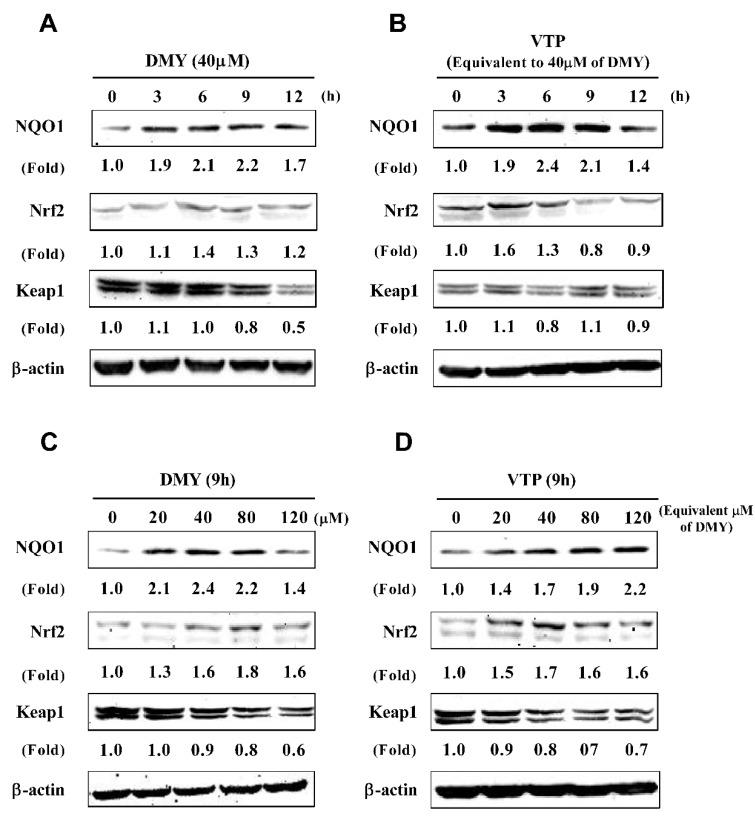
Effects of DMY and VTP on the level of nicotinamide adenine dinucleotide phosphate (NADPH):quinone oxidoreductase (NQO1), nuclear factor erythroid 2-related factor 2 (Nrf2) and Kelch-like ECH-associated protein 1 (Keap1) proteins. A time-effect of DMY (**A**) and VTP (**B**) on the level of NQO1, Nrf2 ,and Keap1 proteins. HepG2 cells were treated with DMY (40 μM) or VTP (equivalent to 40 μM DMY) for 0–12 h. A dose-effect of DMY (**C**) and VTP (**D**) on the level of NQO1, Nrf2, and Keap1 proteins. HepG2 cells were treated with DMY (20–120 μM) and VTP (equivalent to 20–120 μM DMY) for 9 h. The fold was normalized with the control protein, β-actin, and obtained from triplicate blot data.

The results from the *in vitro* data indicated that VTP and its main component DMY possessed antioxidant activity. To clarify whether the antioxidant activity was also observed in the cells, we further investigated the effect of VTP and DMY on the expression of antioxidant enzymes such as NQO1, which is typical antioxidant enzyme in liver and is regulated by the Nrf2/Keap1 pathway. In a time-course experiment, HepG2 cells were treated with 40 μM DMY (Figure 5A) and VTP (equivalent to 40 μM DMY) (Figure 5B) from 0–12 h. Both VTP and DMY enhanced the NQO1 level from 3–12 h, Nrf2 level from 3–6 h, and reduced Keap1 level from 12 h. In a dose-experiment, HepG2 cells were treated with 0–120 μM DMY (Figure 5C) and VTP (equivalent to 0–120 μM DMY) (Figure 5D) for 9 h. Both DMY and VTP enhanced the NQO1 and Nrf2 level from 20–120 μM, and also reduced the Keap1 level in this dose range.

## 4. Discussion

### 4.1. Extraction and Determination of Vine Tea Polyphenols

Vine tea as a traditional herb is widely used in medicine and health supplements in southwest China. A previous study reported that the extraction of tea compounds by ethanol was more efficient than that by water [26]. In order to precisely estimate the chemical and antioxidant properties in vine tea, we optimized the extraction condition by investigating the extraction solvents, solvent concentration, extraction time, and temperature in this study. The extraction condition with 70% ethanol at 70 °C for 40 min with ultrasonic treatment obtained the highest VTP, significantly higher than that by water. Although tea is usually consumed by soaking it into boiling water, there has been an increasing utilization of tea extracts in a variety of foods such as bread, biscuits, and meat products [14,27], and especially in health supplements. Moreover, ethanol is approved as a generally recognized as safe (GRAS) substance by the Food and Drug Administration(FDA) [28]. Furthermore, the extracts of vine tea by 70% ethanol contained a higher total polyphenol content, which seemed to be 2-fold higher than that in green tea, and was 7-fold higher than black tea through a comparison with the results of the polyphenol content in aqueous or ethanol extracts of green tea, black tea, and another 11 leafy herb teas [29]. Thus, the extracts of vine tea by 70% ethanol with ultrasonic treatment will have great potential in efficiently utilizing vine tea.

Generally, tea is a complex mixture containing a range of polyphenols and other components, many of which have well-recognized antioxidant properties [29]. In this study, we found that there was only one major component, DMY, which is as high as 60% in VTP. DMY belongs to flavonoids, which is a special class of phenolic compounds with a structure based on the diphenylpropane carbon skeleton. It is known that DMY is an antioxidant agent in food preservation [30], and also increases antioxidant ability in animal model experiments [31].

### 4.2. Antioxidant Capacity of Vine Tea Polyphenols

Vine tea possesses high polyphenol contents. Accumulated data have indicated that polyphenol content has a significant positive correlation to antioxidant ability [32]. The DPPH free radical assay is an electron transfer reaction, and this assay is rapid and widespread in antioxidant screening. However, the DPPH assay is not a competitive reaction because the small molecules tend to find it easier to bind with the radical site and have a higher value. Meanwhile, the ORAC assay can simulate a human physiological antioxidant situation based on the transfer reaction mechanism. Thus, we used both assays to investigate the antioxidant ability of VTP and DMY. The high antioxidant activity of VTP and DMY were observed in both assays, suggesting that the extracted VTP possessed strong antioxidant capacity and could be developed as an antioxidant agent used in human biology. Moreover, vine tea seems to have higher ORAC value when compared with most consumed green tea ethanol extracts in China [17]. 

The contents of VTP and DMY showed some differences between the three locations. The order of both contents was Guizhou = Hunan > Guangxi, in particular, the DMY content of Guangxi was significantly lower than that from Guizhou and Hunan. We further researched the plantation elevation of the three samples, and found that the plantation elevation of vine tea in Guizhou and Hunan was 800–1300 m, and was 800 m Guangxi. A recent study reported that the antioxidant ability and polyphenol composition were affected by geographic location, growing environment, and leaf grades [18], and black tea contained about 20% more polyphenols when plants were at low elevation. However, our study showed the opposite trend where vine tea from higher plantation elevations contained 30% more polyphenols, and 15% more DMY than those from lower elevations. This result may be due to the different varieties, but this is still the first report and in further study will be required to find the relationship between the growing environment and polyphenolic active compound contents.

Previous studies have reported that DMY could increase antioxidant defense through activation of the ERK and Akt signaling pathways, which induces heme oxygenase-1 expression and thereby protects PC12 cells from H_2_O_2_ induced apoptosis [33,34], and DMY could protect endothelial cells from oxidative stress, and increase the production of nitric oxide [35]. Based on this information, we investigated the effect of VTP and DMY on the Nrf2/Keap1 pathway, which is a master cellular antioxidant defense system against oxidative stress. Our data revealed that VTP and its major component DMY enhanced the level of Nrf2, a positive factor for the Nrf2/Keap1 pathway, and reduced the level of Keap1, a negative factor for the Nrf2/Keap1 pathway. Sequentially, the level of downstream antioxidant enzyme, NQO1, was increased by VTP and DMY in a dose- and time-dependent manner. These data demonstrated that vine tea and its major compound DMY might exert an antioxidant activity in culture cells by activating the Nrf2/Keap1 pathway. 

## 5. Conclusions

In conclusion, the extraction of vine tea by 70% ethanol with ultrasonic treatment is a novel method to efficiently obtain the bioactive components that possess stronger DPPH scavenging ability and ORAC *in vitro*. Moreover, they enhanced the level of the antioxidant enzyme, NQO1, in culture cells by activating the Nrf2/Keap1 pathway. These findings will help us understand the mechanism of the health function of a traditional vine tea.
